# 61586 Impact of Diabetes and Pre-diabetes on Prevalence of Infection with Mycobacterium tuberculosis among Household Contacts of Active Tuberculosis Cases in Ethiopia

**DOI:** 10.1017/cts.2021.483

**Published:** 2021-03-30

**Authors:** Alison G.C.S. Smith, Russell R. Kempker, Liya Wassie, Kidist Bobosha, Azhar Nizam, Neel Gandhi, Matthew J. Magee, Henry M. Blumberg

**Affiliations:** 1 Emory University School of Medicine; 2 Emory University Department of Medicine; 3 Armauer Hansen Research Institute, Addis Ababa, Ethiopia; 4 Emory University Rollins School of Public Health

## Abstract

ABSTRACT IMPACT: This work examines the association between diabetes mellitus and latent tuberculosis infection among a cohort of household contacts exposed to active tuberculosis in Ethiopia, focusing attention on the need for further translational research to determine the mechanisms of susceptibility to Mycobacterium tuberculosis infection among individuals with diabetes and pre-diabetes. OBJECTIVES/GOALS: Diabetes mellitus (DM) is an established risk factor for active TB disease, but there is limited understanding of the relationship of DM and latent tuberculosis (LTBI). We sought to determine the relationship between DM or pre-DM with LTBI among household or close contacts (HHCs) of active TB cases in Ethiopia. METHODS/STUDY POPULATION: We conducted a cross-sectional study of the HHCs of index active TB cases enrolled in an ongoing TB Research Unit (TBRU) study in Addis Ababa, Ethiopia. HHCs of individuals with laboratory-confirmed TB had QuantiFERON ®-TB Gold Plus (QFT) and glycated hemoglobin (HbA1c) tests performed. LTBI was defined as a positive QFT and lack of symptoms. HbA1C results were used to define no DM (HbA1c <5.7), pre-DM (HbA1c 5.7-6.5%), and DM (HbA1c >6.5% or prior history of diabetes). Logistic regression was used to estimate adjusted odds ratios (OR) and 95% confidence intervals (CI) after adjustment for age, sex and HIV status as potential confounders. RESULTS/ANTICIPATED RESULTS: Among 466 HHCs, the median age was 29 years (IQR 23-38), 58.8% were female, 3.4% were HIV-positive, and median BMI was 20.9 kg/m^2 (IQR 18.9-23.8). Overall, 329 HHCs (70.6%) had LTBI, 26 (5.6%) had DM and 73 (15.7%) had pre-DM. Compared to HHC without DM, the prevalence of LTBI was higher in those with pre-DM (68.9% vs. 72.6%; OR 1.19, 95% CI 0.69-2.13) and those with DM (88.5%; OR 3.45, 95% CI 1.17-14.77). In multivariable analysis, there was a trend towards increased LTBI risk among HHCs with DM vs. without DM (OR 2.16, 95% CI 0.67-9.70) but the difference was not statistically significant. Among HHCs with LTBI, the median IFN-? response to TB1 antigen was modestly greater in those with DM (5.3 IU/mL; IQR 3.0-7.8) and pre-DM (5.4 IU/mL; IQR 2.0-8.4) compared to HHCs without DM (4.3 IU/mL; IQR 1.4-7.7). DISCUSSION/SIGNIFICANCE OF FINDINGS: Our results suggest that DM may increase the risk of LTBI among HHCs recently exposed to active TB. Among those with LTBI, increased IFN-? antigen response in the presence of DM and pre-DM may indicate an exaggerated but ineffectual response to TB. Further investigation is needed to assess how dysglycemia impacts susceptibility to M. tuberculosis.

